# Prognostic role of RDW in hematological malignancies: a systematic review and meta-analysis

**DOI:** 10.1186/s12935-018-0558-3

**Published:** 2018-04-23

**Authors:** Lisha Ai, Shidai Mu, Yu Hu

**Affiliations:** Institute of Hematology, Union Hospital, Tongji Medical College, Huazhong University of Science and Technology, Wuhan, 430022 China

**Keywords:** Red blood cell distribution width, Hematologic malignances, Prognosis, Meta-analysis

## Abstract

**Background:**

Red blood cell distribution width (RDW), a biomarker for discrimination of anemia, has been recently identified as a prognostic factor in various types of cancer. Here we performed a meta-analysis in order to assess the correlation between RDW and the survival outcomes in patients with hematologic malignances.

**Patients/methods:**

We systematically searched PubMed, Embase, and ISI Web of Science for relevant studies, to investigate the prognostic significance of RDW in hematological malignancies. Odds ratios or hazards ratios (HRs) with corresponding 95% confidence intervals (CIs) are pooled to estimate the association between RDW and clinicopathological parameters of patients with hematologic malignances.

**Results:**

Seven trials with 1031 patients suffering from hematological malignancies were included in the meta-analysis, and the results indicated that increased pretreatment RDW predicted poor overall survival (HR = 2.35, 95% CI 1.70–3.24), poor progress-free survival (HR = 2.44, 95% CI 1.70–3.49) and poor event-free survival (EFS) (HR = 3.15, 95% CI 1.59–6.25). Furthermore, the similar results were observed in subgroup analysis stratified by cancer type, such as multiple myeloma, and diffuse large B cell lymphoma, etc.

**Conclusions:**

As for hematologic malignances, patients with higher RDW are more likely to have poorer prognosis than those with lower RDW.

## Background

Hematological malignancies mainly include leukemia, lymphoma, and plasma cell neoplasm. There were about 172,910 new cases of hematological malignancies and 58,300 deaths due to hematological malignancies projected to occur in 2017 in USA [1]. Great advances have recently been achieved in the therapy for patients with hematologic malignances. However, the overall survival for patients has not been obviously improved. Identification of prognostic factors for hematologic malignancies is very helpful for clinicians to choose therapeutic strategies and for patients to improve their prognosis.

A number of prognostic molecular markers for hematologic malignances have been identified, however, many of these prognostic means are costly, difficult to perform, or not easily interpreted. Therefore, other prognostic models that are inexpensive, widely available, and easily interpreted are urgently needed for clinicians.

Red blood cell distribution width (RDW) is a parameter measured in blood routine test, and is widely used to distinguish between different types of anemia [2]. As an easy-to-measure marker of the systemic inflammatory response, the RDW has been established as a novel prognostic factor in many pathophysiological conditions, including cardiovascular disease [3, 4] and inflammation [5, 6]. Recently, RDW grows to be recognized as an independent prognosis factor in numerous types of cancer, including lung cancer [7], gastrocolorectal cancer [8], breast cancer [9], prostate cancer [10], as well as in several types of hematologic malignances.

However, due to the variance in the study design and sample size, direct impact of RDW level on hematologic malignances patients’ survival remains inconclusive. In this study, we searched PubMed (Medline), OVID (Embase), and ISI Web of Science databases for relevant studies and performed a meta-analysis in order to assess the correlation between RDW and the survival outcomes in patients with hematologic malignances.

## Methods

### Search strategy

We conducted the systematic search strategies described by Dickersin et al. [11] to identify all relevant electric publications until January 2018 throughout databases, including (Medline), OVID (Embase), and ISI Web of Science databases. The search strategy included terms are as follow: “RDW” (e.g. “red blood cell distribution width”), “prognosis” (e.g. “outcome” “survival” “mortality” “recurrence” “progression” “metastasis”) and “hematologic malignancies” (e.g. “leukemia” “lymphoma” “myeloma” “myelodysplastic syndromes”). Furthermore, we manually checked the reference lists of retrieved studies to identify more potential pertinent studies.

### Selection criteria

Studies were included in the meta analysis if they met all of the following criteria: (i) patients were diagnosed with hematologic malignancies; (ii) association between the pretreatment RDW and OS, PFS or other clinicopathological parameters was reported; (iii) studies that were not directly reporting hazard ratios (HRs) and 95% CI were allowed if we could reconstruct them by *p* values and other data reported [12]; (iv) the publication language was confined to English. Exclusion criteria were: (i) abstracts, letters, reviews, case reports, etc.; (ii) studies with insufficient data for analysis; (iii) studies without specific data concerning hematologic malignancies or RDW; (iv) multiple published reports. When there were several reports concerning the same cohort, we included the most recent publication in our meta-analysis.

### Data extraction

Two investigators (Lisha Ai and Shidai Mu) independently identified the eligible studies for this meta-analysis. Any disagreement was resolved by discussion with the other researcher (Yu Hu). The qualities of the included studies were assessed according to the Newcastle–Ottawa Quality Assessment Scale (NOS) [13]. This scale uses a star system (with a maximum of nine stars) to evaluate a study in three domains: selection of participants, comparability of study groups, and the ascertainment of outcomes of interest. NOS scores of ≥ 7 were assigned as high-quality studies.

For each study, the following relevant data were extracted in a predefined table: (i) first author’s name, year of publication, country of the population, sample size, patient age, follow-up period; (ii) survival data including overall survival (OS), progression free survival (PFS) and event free survival (EFS) (OS was calculated from the medical treatment until the death of patient or the last follow-up. PFS was defined as the interval between the date of treatment and the detection of the recurrence tumor or death from any cause. EFS was calculated from the first day of diagnosis until any events, such as disease progression or relapse, initiation of another treatment, death due to any cause, etc.); Get Data Graph Digitizer (http://getdata-graph-digitizer.com/) were used to obtain the data from the survival curve. (iii) cut-off value used to define “elevated RDW”.

### Statistical analysis

Hazard ratio (HR) and 95% confidence intervals (95% CI) were obtained directly from each literature or from estimation according to the methods by Parmer et al. [12]. Heterogeneity among included studies was checked by the χ^2^-based Q test and I^2^ test [14]. The fixed-effect model was used for analysis without any significant heterogeneity between studies (*p *> 0.10, I^2^ < 50%). Otherwise, the random-effects model was chosen. Subgroup analysis was further performed to explore the source of heterogeneity. Sensitivity analysis was also performed to examine the effect of each study on the overall pooled results. Publication bias was evaluated by using Begg’s test and Egger’s test. Trim-and-fill method was employed to further assess the possible effect of publication bias [39]. All analyses were carried out using STATA statistical software package version 12.0 (STATA, College Station, TX).

## Results

### Selection and characteristics of included studies

**Fig. 1:**
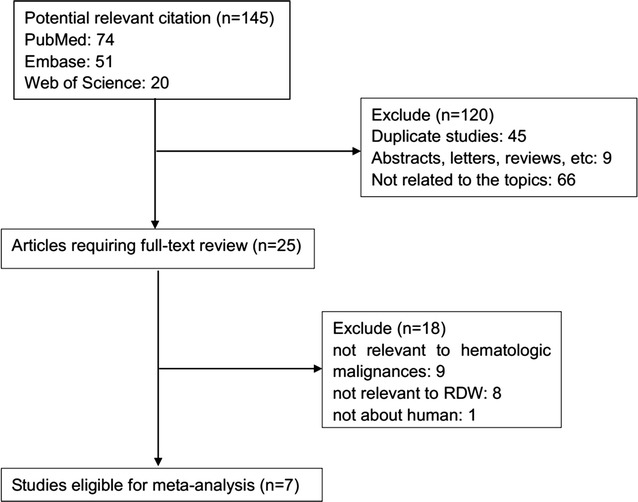
Flow diagram of selecting relevant studies included in the meta-analysis

As shown in Fig. 1, the initial search algorithm retrieved a total of 145 studies. After excluding the duplicates (n = 45); abstracts, letters, reviews, etc. (n = 9); and the studies not related to research topics (n = 66), the remaining studies (n = 25) were further reviewed by reading the full text. Additional 18 studies were then excluded because they didn’t provide specific data concerning hematologic malignances nor RDW. Therefore, 7 studies between 2014 and 2017 with a total 1031 hematologic malignances patients were enrolled in our meta-analysis.

Summary on the characteristics of the included studies were shown in Table 1. These studies were from China, Japan, Korea and Croatia, which evaluated several type of hematologic malignancies, including three for multiple myeloma (MM), two for diffuse large B cell lymphoma (DLBCL), one for extranodal NK/T lymphoma (ENKT), and one for chronic myeloid leukemia (CML). Five studies enrolled > 100 patients and two studies had < 100 patients. The cutoff value defining high RDW in these studies was not uniform and ranged from 14.0 to 18.05. One study used RDW-SD (standard deviation) for RDW and others used RDW-CV (covariance). 885 patients from six studies reported OS, 664 patients from four studies reported PFS and 171 patients from two studies reported EFS. Six studies directly reported HR and 95% CI in the original literature. NOS score was above 7 in 4 studies.

**Table 1:** Characteristics of studies included in the meta-analysis

Study	Year	Country	Cancer types	Sample size	Cut-off	Age	Follow-up (month)	Survival analysis	HR	NOS score
Zhou	2017	China	DLBCL	161	14.1	59 (18–80)	42 (6–120)	OS, PFS	Reported	7
Wang	2017	China	MM	196	18.05	65 (33–82)	33.5 (1–120)	OS	Reported	7
Meng	2017	China	MM	166	14	61.6	17.79 (0.63–62.83)	OS, PFS	Reported	4
Luo	2017	China	NK/T lymphoma	191	46.2^a^	44 (15–86)	30 (2–97)	OS, PFS	Reported	7
Perisa	2015	Croatia	DLBCL	81	15	64	NR	OS, EFS	Reported	5
Iriyama	2015	Japan	CML	90	15	NR	168	OS, EFS	Estimated	5
Lee	2014	Korea	MM	146	14.5	61 (32–83)	120	PFS	Reported	6

*NR* not reported, *NOS* Newcastle–Ottawa Quality Assessment Scale

^a^RDW was present as RDW-SD

### Association between RDW and survival of hematologic malignances patients

**Fig. 2:**
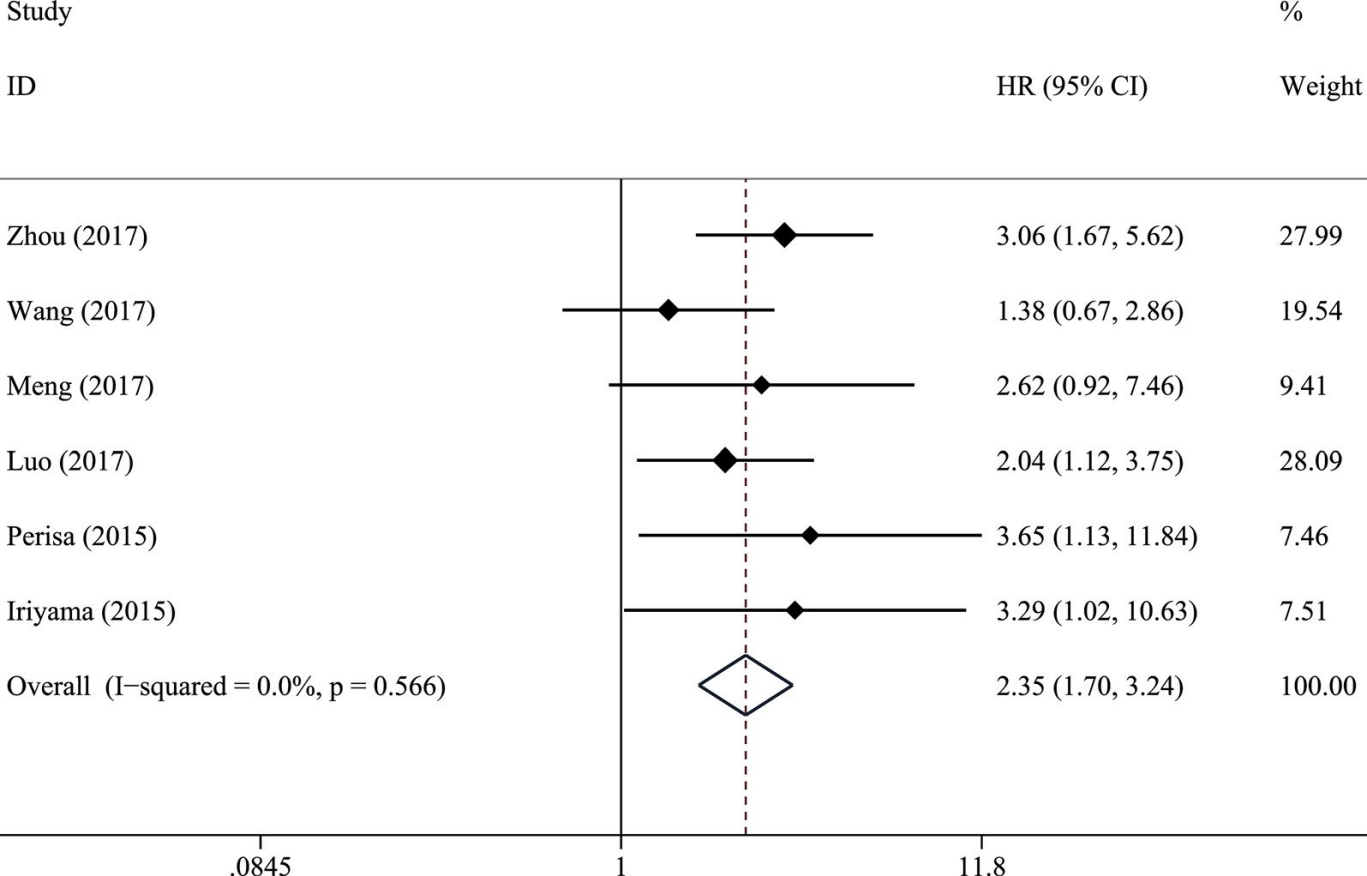
Forest plot for the association between elevated RDW and OS in hematologic malignances

**Fig. 3:**
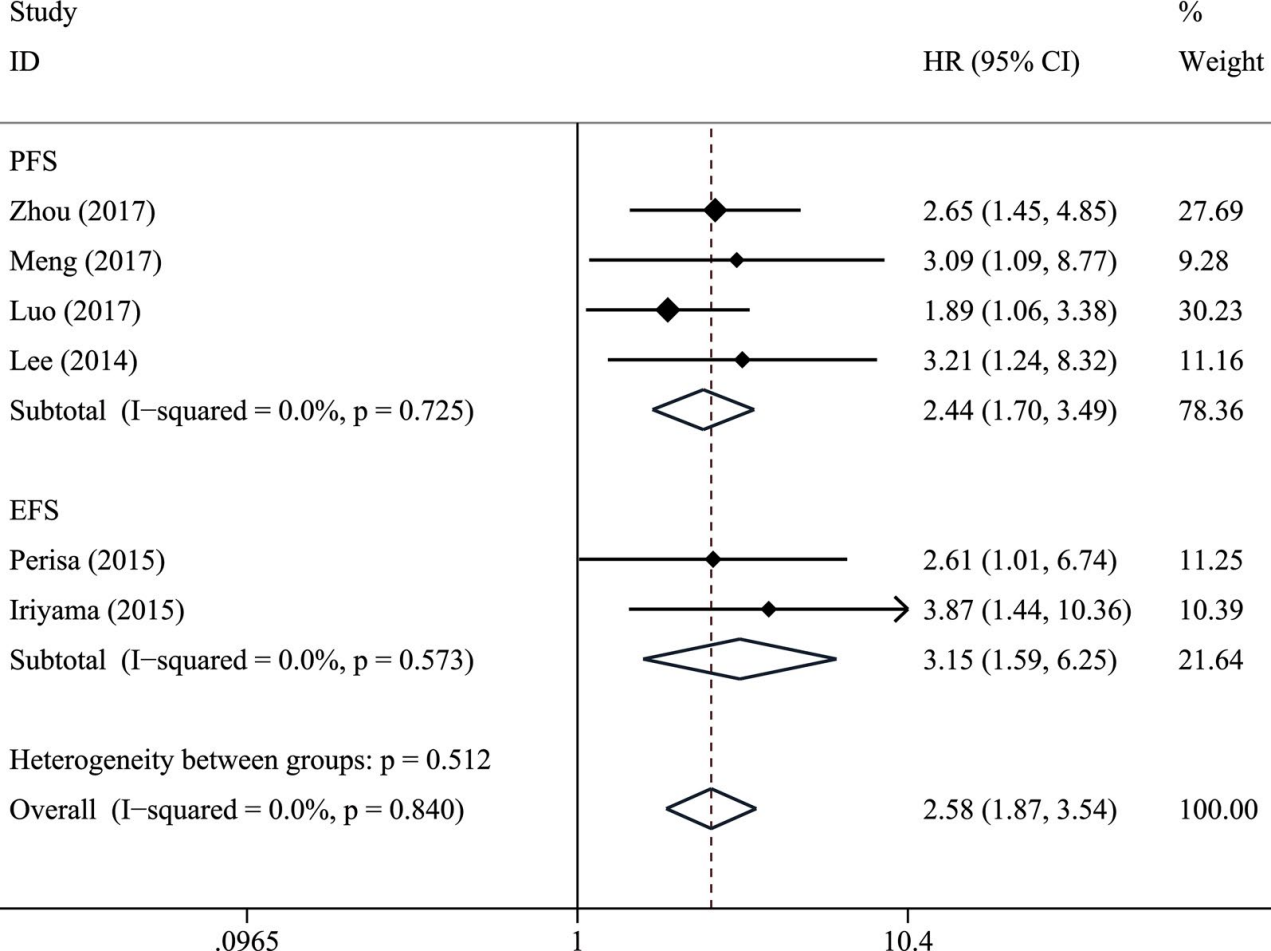
Forest plot for the association between elevated RDW and PFS and EFS in hematologic malignances

7 studies in our analysis examined the association between RDW and survival of patients with hematologic malignances. As shown in Fig. 2, the combined results of 7 studies showed elevated RDW was associated with poor OS (HR = 2.35, 95% CI 1.70–3.24) without significant heterogeneity (I^2^ = 0%, P_heterogeneity_ = 0.566). Figure 3 summarized HR for PFS (HR = 2.44, 95% CI 1.70–3.49) and EFS (HR = 3.15, 95% CI 1.59–6.25), and there were no heterogeneity between the studies (I^2^ = 0%, P_heterogeneity_ = 0.725; and I^2^ = 0%, P_heterogeneity_ = 0.573, respectively).

**Fig. 4:**
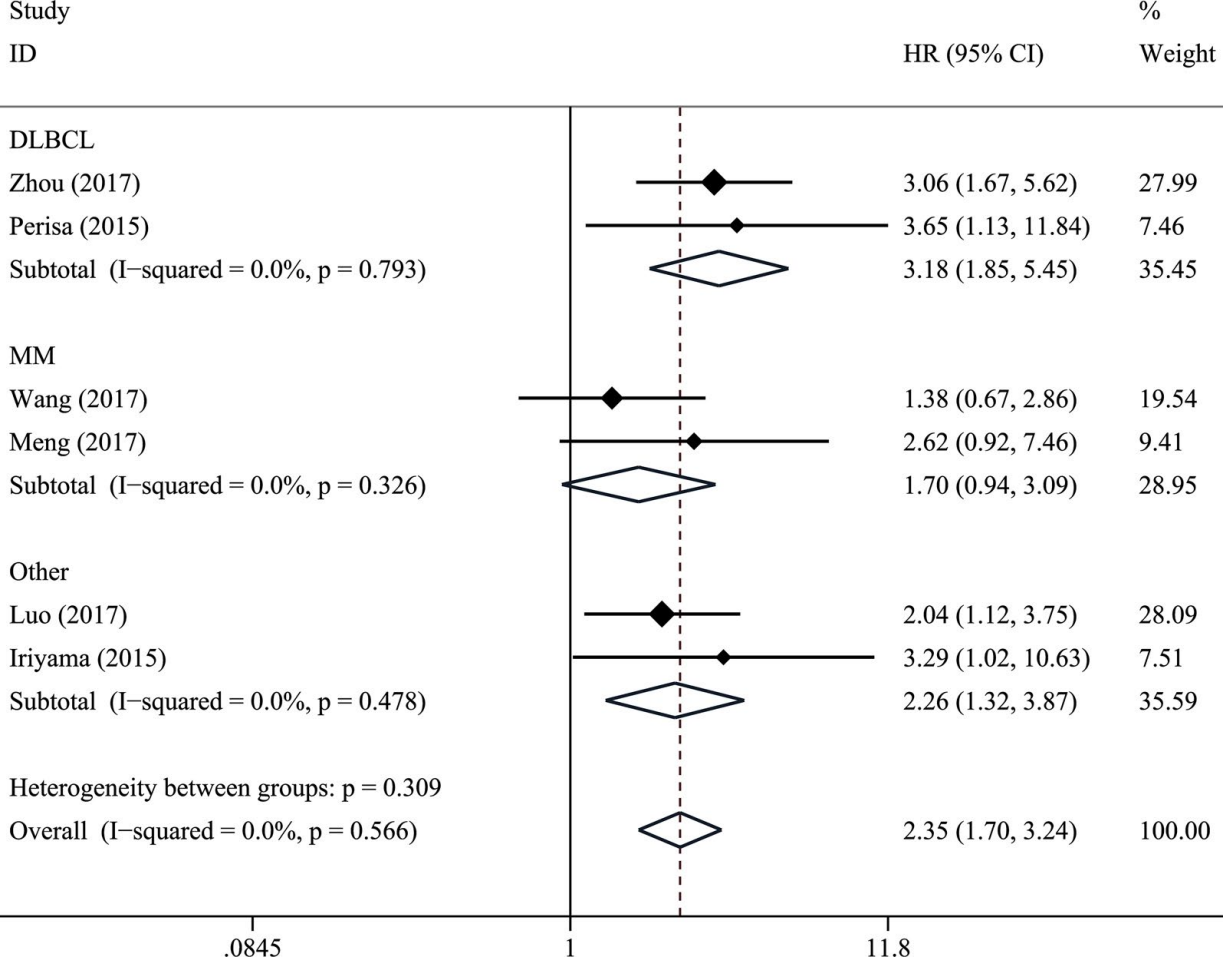
Forest plot for the association between RDW and OS in different types of hematologic malignances

Subgroup analysis for OS was also performed stratified by cancer type. As shown in Fig. 4, summarized HR for DLBCL (HR = 3.18, 95% CI 1.85–5.45), MM (HR = 1.70, 95% CI 0.94–3.09) and other types (HR = 2.26, 95% CI 1.32–3.87), and there was no heterogeneity between the studies (I^2^ = 0%, P_heterogeneity_ = 0.793; I^2^ = 0%, P_heterogeneity_ = 0.326; and I^2^ = 0%, P_heterogeneity_ = 0.478, respectively).

### Sensitivity analysis

**Fig. 5:**
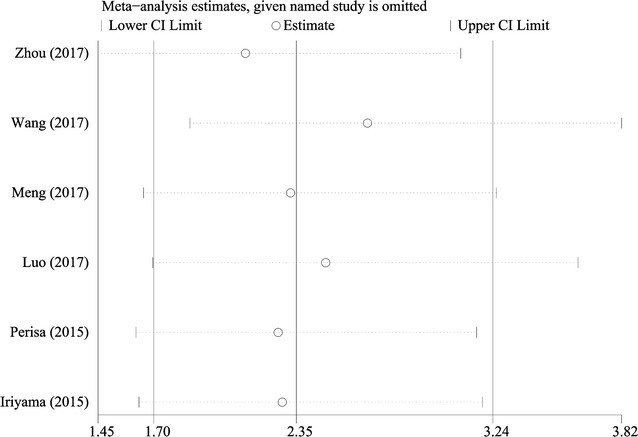
Sensitivity analysis of the enrolled analysis

Sensitivity analyses were performed next. A single study involved in the meta-analysis was deleted each time to unveil the influence of the individual data set on the pooled HRs. As shown in Fig. 5, there was no study obviously impacting the combined results, which indicated the robustness of our meta-analytic results.

### Publication bias

**Fig. 6:**
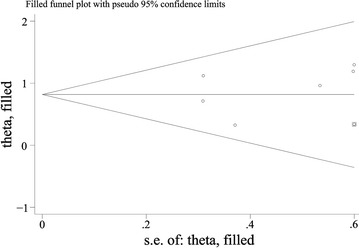
Funnel plot with trim and fill

To assess publication bias in this study, the included studies were conducted by using Begg’s funnel plots and Egger’s test. The results indicated the possibility of publication bias among the studies included in our analysis (*p *= 0.481). Therefore, “trim and fill” analysis was further performed, and the result indicated that one relevant study evaluating the prognostic value of elevated RDW in hematologic malignances patients remained unpublished. However, the pooled HR of 2.27 (95% CI 1.66–3.09) obtained from trim and fill method was statistically significant with a symmetrical funnel plot (Fig. 6).

## Discussion

Cancer associated inflammation is recognized as a hallmark feature of tumor development and progression. Previous studies have reported the association between RDW and the clinical outcome of solid tumor. Recently, numerous studies have provided evidence on the correlation between elevated RDW and poor prognosis in hematologic malignances, including chronic lymphocytic leukemia (CLL) [15], chronic myeloid leukemia (CML) [16], DLBCL [17, 18], NK/T lymphoma [19], as well as multiple myeloma [20–22].

However, these results are not comparable, because of the heterogeneous designs and patient population, and the diversity in cut-off value defining “elevated RDW”. Our study is the first meta-analysis covering a total of 7 published studies with 1031 patients to clarify the prognostic value of RDW in the pretreatment patients with hematologic malignances. The combined results indicated that elevated RDW significantly predicted poor OS, poor EFS and poor PFS of patients with hematologic malignances. Furthermore, the similar results were observed in subgroup analysis stratified by cancer type, such as MM, DLBCL, etc.

The prognostic value of RDW was investigated in a variety of cancer patients and gathering evidences suggested that RDW was an independent factor for prognosis [23, 24]. The exact mechanism underlying the associations of RDW with these cancers has not been clearly elucidated. Recently, numerous studies have reported the positive correlation between RDW and a variety of inflammatory markers, including the erythrocyte sedimentation rate (ESR), interleukin-6 (IL-6), C-reactive protein (CRP), soluble tumor necrosis factor (TNF) receptors I and II, and soluble transferrin receptor [25]. One possible explanation for this finding is that inflammation impairs erythropoiesis and causes changes in red blood cell maturation, which contributed to the increase in RDW [26]. In addition, RDW was found to be associated with malnutrition (i.e., deficiencies in nutrients such as vitamin B12 and folate), which has been shown to be correlated to lower response to treatment, and poorer prognosis in cancer patients [18]. Moreover, in the terminal stage of malignancy, digestive system dysfunction may lead to inadequate resorption of the iron, resulting in the disturbed iron metabolism and the inhibition of iron transport in the blood, which might contribute to increased RDW levels. This mechanism has been found in most of the cancers [27]. Therefore, elevated RDW might bridge the relationship between inflammation and tumorigenesis, thereby correlating to poor prognosis of cancer patients.

This meta-analysis had some limitations that call for cautious interpretation of the results. First, only 7 studies were included in this meta-analysis, and tumor types of this study were also limited, which could decrease the accuracy of the results. Second, the cut-off value defining elevated RDW varied among studies (Table 1). Third, differences of paper quality and sample size across the studies might cause bias in the meta-analysis. Forth, most of the included studies reported positive results, therefore our results might overestimate the prognostic significance of RDW to some degree.

Despite the above limitations, our meta-analysis supports the values of RDW for predicting survival outcome in patients of hematologic malignances. RDW can be easily obtained from routine blood tests, thus intermediate assessments about changes in RDW during therapy were simply available. That is, RDW can help personalize the treatment intensity, as well as aftercare schedule, in order to increase the likelihood of early detection.

## Conclusion

Here, we searched electronic databases for relevant studies, and enrolled 7 studies with a total of 1031 patients for meta-analysis, drawing a conclusion that patients with higher RDW are more likely to have poorer prognosis than those with lower RDW. Taken together, the results from our meta-analysis suggest that RDW gains a prognostic value for patients with hematologic malignances. More multi-center prospective cohorts should be conducted to further validate the role of the RDW in hematologic malignances.

## Abbreviations

RDW: red blood cell distribution width
OR: odds ratio
HR: hazards ratios
CI: confidence interval
OS: over survival
PFS: progress-free survival
EFS: event-free survival
NOS: Newcastle–Ottawa Quality Assessment Scale
MM: multiple myeloma
DLBCL: diffuse large B cell lymphoma
ENKT: extranodal NK/T lymphoma
CML: chronic myeloid leukemia
SD: standard deviation
CV: covariance
CLL: chronic lymphocytic leukemia
ESR: erythrocyte sedimentation rate
IL-6: interleukin-6
CRP: C-reactive protein
TNF: tumor necrosis factor
